# Nutrition and Survival of 150 Endoscopic Gastrostomy-Fed Patients with Amyotrophic Lateral Sclerosis

**DOI:** 10.3390/nu17081292

**Published:** 2025-04-08

**Authors:** Diogo Sousa-Catita, Paulo Mascarenhas, Cátia Oliveira, Miguel Grunho, Carla A. Santos, João Cabrita, Paula Correia, Jorge Fonseca

**Affiliations:** 1 Aging Lab, Egas Moniz Center for Interdisciplinary Research (CiiEM), Egas Moniz School of Health & Science, Caparica, 2829-511 Almada, Portugal; pmascarenhas@egasmoniz.edu.pt (P.M.); miguelgrunho@gmail.com (M.G.); jorgedafonseca@gmail.com (J.F.); 2 Residências Montepio, Serviços de Saúde, SA.Rua Julieta Ferrão N° 10–5°, 1600-131 Lisboa, Portugal; 3 APELA—Portuguese Association of Amyotrophic Lateral Sclerosis, 1900-221 Lisboa, Portugal; joao.cabrita@apela.pt; 4 Egas Moniz Center for Interdisciplinary Research (CiiEM), Egas Moniz School of Health & Science, Caparica, 2829-511 Almada, Portugal; 5 GENE—Artificial NutritionTeam, Department of Gastroenterology Hospital Garcia de Orta, 2805-267 Almada, Portugal; sofi.doliveira@gmail.com (C.O.); carla.adriana.santos@hotmail.com (C.A.S.); 6 Department of Neurology, Hospital Garcia de Orta, 2805-267 Almada, Portugal; 7 Department of Otorhinolaryngology, Hospital Garcia de Orta, 2805-267 Almada, Portugal; paulacorreia.tf@gmail.com

**Keywords:** amyotrophic lateral sclerosis, nutritional status, percutaneous endoscopic gastrostomy, survival

## Abstract

**Background/Objectives**: Amyotrophic lateral sclerosis (ALS) is a progressive neurodegenerative disorder affecting motor neurons in the brain and spinal cord, leading to muscle weakness, atrophy, and paralysis. Treatment focuses on symptom management, using medication, physiotherapy, and nutritional support. In this context, endoscopic gastrostomy (PEG) can provide adequate feeding, hopefully improving nutrition and preventing complications. **Methods**: We studied ALS patients undergoing PEG over three months post-procedure, using anthropometry ((BMI)—body mass index; (MUAC)—mid-upper arm circumference; (TSF)—tricipital skinfold; (MAMC)—mid-arm muscle circumference) and laboratory data (Albumin; Transferrin; total cholesterol and hemoglobin), evaluating survival, complications, and nutritional/clinical status. Statistical analysis included Kaplan–Meier survival estimation and Cox regression to assess nutritional markers associated with survival. **Results**: 150 ALS patients underwent gastrostomy, mostly older adults (mean age: 66.1 years; median: 67). Mean survival was 527 [95% CI: 432–622] days, median 318 [95% CI: 236–400]. ALS bulbar subtype, MUAC and MAMC positively impacted PEG-feeding survival time (*p* < 0.05, Wald test). During the first three months of PEG feeding, each unit increase (cm) in MUAC and MAMC lowered death risk by 10% and 11%, respectively, highlighting the importance of nutrition care for survival. The bulbar subtype showed higher PEG feeding survival, with a 55.3% lower death hazard than the spinal subtype. There were no major PEG complications. **Conclusions:** ALS patients present a high risk of malnutrition. Patients that improved MAMC and MUAC in the first three PEG-fed months presented longer survival. Early PEG nutrition, even when some oral feeding is still possible, may reinforce the preventative role of enteral feeding in maintaining nutrition and potentially improving survival.

## 1. Introduction

Amyotrophic lateral sclerosis (ALS), also known as Lou Gehrig’s disease in the United States of America and Charcot’s disease in France, is a progressive neurodegenerative disorder affecting nerve cells in the brain and spinal cord, specifically targeting the motor neurons responsible for voluntary muscle control, thus hampering several motor functions, including walking, talking, swallowing, and breathing [1]. As the disease advances, motor neurons degenerate and die, resulting in muscle weakness, fasciculations, muscle atrophy, and eventually paralysis. Early symptoms may mainly include limb weakness, fasciculations or muscle cramps in the spinal clinical phenotype, or speech and swallowing difficulties in the bulbar form. Progression of ALS can lead to respiratory impairment, ultimately causing respiratory failure and death [2].

The etiology of ALS remains incompletely understood, although a combination of genetic and environmental factors is believed to contribute to the disease’s development. While there is currently no cure for ALS, treatments aim to increase life span, manage symptoms, and enhance the quality of life throughout the survival period. These may include medications (e.g., riluzole), nutritional support, physical therapy, and assistive devices for breathing, mobility, and communication [2,3]. Disease progression differs among individuals, with some experiencing a rapid decline while others have a more gradual course. Prognosis is generally poor, with a survival time of 2–5 years from symptom onset, although there are rare instances of people living with ALS for over 20 years [4].

Collaboration among healthcare providers is crucial for individuals with ALS, not only to manage symptoms but also to optimize their quality of life. Support from family, friends, and support groups can be beneficial in coping with the challenges associated with the disease [3,5]. Due to the progressive muscle weakness and atrophy, swallowing difficulties, respiratory problems, and cognitive changes associated with ALS, the disease has a significant impact on nutritional status, making it difficult to maintain weight and lean mass [1,2,3,4,5].

Adequate nutrition is essential for people with ALS, as it supports muscle function, immune responses, and wound healing. Nutritional interventions, such as dietary modifications and the various forms of enteral nutrition, can help to maintain adequate nutrition and enhance overall health and quality of life [3,5]. A team of dietitians, nurses, speech therapists, gastroenterologists, and neurologists must collaborate with ALS patients to develop personalized nutrition plans that address their needs and tackle any challenges they may face while eating and drinking. Regular monitoring of the nutritional status facilitates early identification and intervention [5].

Percutaneous endoscopic gastrostomy (PEG) is a medical procedure involving the insertion of a feeding tube directly into the stomach through a small abdominal incision. PEG may be recommended for ALS patients experiencing swallowing difficulties or are at risk of malnutrition or dehydration. By providing direct access to the stomach, PEG enables enteral nutrition administration, preventing malnutrition and supporting the preservation of muscle mass [3]. It may also help to reduce the risk of aspiration pneumonia, a common concern for ALS patients with swallowing difficulties [1,5]. PEG has been reported in several studies as beneficial for improving the quality of life and prolonging survival in ALS [6,7,8]. PEG can be performed in these patients under procedural non-invasive ventilation with minimal peri- and post-procedural complications. In addition, data showed no significant difference in long-term survival rate among patients with high (>50%) and low (<50%) forced vital capacity (FVC). This finding was in contrast to the results of other studies showing a lower survival rate after PEG tube placement in patients with ALS who had low FVC (<50%) [9]. The decision to proceed with PEG should be carefully planned, considering each individual’s specific circumstances, and must be consensual for patients, their caregivers, and their healthcare team [1,4,5,6,10]. In fact, for individuals diagnosed with ALS who experience dysphagia, it is recommended to insert a PEG tube as soon as possible, ideally before the onset of malnutrition and respiratory complications. This approach ensures the safety and effectiveness of the procedure, thereby enhancing patient outcomes [1,7,11].

### Objectives

The study aims to evaluate the clinical impact and safety of PEG tube placement and feeding in ALS patients. To achieve this goal, the research will characterize the clinical and nutritional status of ALS patients at the time of PEG tube placement (baseline) and at three-month follow-ups, using readily accessible assessment tools such as anthropometric measurements and laboratory biomarkers. It will also investigate the relationship between patient survival and clinical/nutritional status at both baseline and follow-up, considering overall survival and differences across age groups, sex, and ALS clinical subtypes (spinal vs. bulbar). Furthermore, the study will determine the safety profile of PEG tube placement and feeding by analyzing the occurrence of major procedure- or feeding-related complications. Finally, it will analyze the temporal dynamics between symptom onset, ALS diagnosis, and PEG tube placement, evaluating their association with nutritional status and survival across different patient subgroups, including variations by ALS subtype, age, sex, and short- versus long-term survival.

## 2. Materials and Methods

### 2.1. Patients

We studied consecutive adult patients with ALS, according to the revised El Escorial [12] or the Awaji criteria [13], who were referred for and underwent endoscopic gastrostomy for nutritional support from January 2001 to July 2023. Patients with ALS were considered eligible if they were referred to PEG by their attending clinicians. All data are part of the routine evaluation of PEG patients and were collected from the GENE (Hospital Garcia de Orta Artificial Nutrition Team) clinical files.

All ALS patients referred to our Artificial Nutrition Team files were eligible for the study, although those without the clinical conditions necessary for PEG, which precluded the procedure, or with insufficient data in their clinical files, were excluded.

### 2.2. Variables

ALS clinical subtype: Based upon the classification and/or the clinical information (description of the regional distribution of motor involvement at onset) provided by the referring physician, the patients were classified as having either spinal ALS (motor onset in the upper or lower limbs) or bulbar ALS (motor onset in the bulbar controlled muscles). Due to the heterogeneity of the clinical background and/or differentiation of the referring physician (ranging from the hospital doctor to the neurologist specialized in neuromuscular disorders) and of the classification/clinical information recorded on the referrals, no further classification was attempted, including the categorization according to more specific regional subtypes or based on relative upper motor neuron versus lower motor neuron [14,15].

Clinical outcome: We collected the survival time (in days) of the PEG-fed ALS patients, starting from the day of the PEG procedure up to the time of death, or until 31 July 2023. When available, we recorded the date of the appearance of the first symptoms and the date of diagnosis.

Minor complications with PEG are frequent and easy to solve. However, major complications such as aspiration pneumonia, major hemorrhage, buried bumper syndrome, perforation of the bowel, necrotizing fasciitis, stoma infection, and tube displacement [10] are a significant concern, and our team aimed to ensure the safety of this procedure, both immediately and later on during the follow up. We recorded all potential major complications associated with PEG during the follow-up.

Anthropometric evaluation: We recorded clinical and anthropometric data on the endoscopic gastrostomy day (T0) and three months after endoscopic gastrostomy (T3). The anthropometric measurements followed the International Society for the Advancement of Kinanthropometry manual. Three consecutive measurements were obtained, and the mean value of those measurements was recorded.

Body mass index (BMI): BMI was obtained in most patients using the equation Weight/Height^2^. If patients were bedridden and could not stand up for weight and height evaluation, BMI was estimated using the mid-upper arm circumference (MUAC) and regression equations described by Powell-Tuck and Hennessy [16]. This method has been previously used and proved to provide reliable BMI estimation in PEG patients [17]. Each patient was graded following the Lipschitz classification according to age [18] (Table 1).

MUAC was evaluated using an inextensible measuring tape with a 1 mm resolution. MUAC evaluates several arm tissues, representing both body fat and lean mass. Tricipital skinfold (TSF) was measured using a Lange Skinfold caliper with a 1 mm resolution. TSF evaluates the subcutaneous adipose tissue and estimates adipose tissue reserves. The Mid-Arm Muscle Circumference (MAMC) was calculated according to the equation: MAMC = MUAC (cm) − 0.314 × TSF (mm). The MAMC allows us to estimate lean and muscle mass. For each patient, MUAC, MAMC, and TSF were compared with reference values from the National Health and Nutrition Examination Survey (NHANES) through the comparison with the Frisancho reference tables [19,20]. Although nutritional evaluation could benefit from more sophisticated devices for measuring body composition, such as bioelectrical impedance analysis (BIA) or CT Scan analysis, those devices were not available for all patients in all clinical settings. Although less precise, BMI and anthropometric measures, classically used to evaluate fat/lean mass [21], are inexpensive and widespread nutritional evaluation tools available everywhere, even in institutions with scarce resources.

Laboratory evaluation: A blood sample was taken minutes before the endoscopic gastrostomy procedure (T0) and three months after the gastrostomy procedure (T3). Blood samples were obtained between 8:00 a.m. and 10:00 a.m., following at least 12 h of fasting. Serum albumin < 3.5 g/dL, serum transferrin < 200 mg/dL, serum total cholesterol < 160 mg/dL, and hemoglobin < 13 g/dL for males or <12 g/dL for females were considered low values, suggestive of poor prognosis and malnutrition [22,23,24]. Nevertheless, laboratory data were always regarded as dependent on several non-nutritional influences.

### 2.3. PEG Tube Placement Procedure

Gastrostomies were performed on an outpatient basis using the pull technique, with 20Fr kits. Patients were sedated by an anesthetist using personalized doses of propofol. After the patients were admitted to the endoscopy unit, blood tests and clinical/anthropometric data were collected. The patients remained in the recovery room until stable enough to be discharged from the endoscopy unit. At the end of the procedure, patients were reevaluated between the third and fourth day after gastrostomy to confirm adequate healing and use of the enteric access.

### 2.4. Ethical Considerations

All patients were informed about the procedures of the Artificial Nutrition Team for PEG-feeding patients and gave their informed consent. The Hospital Garcia de Orta Ethics Committee approved this retrospective study, and it was conducted in accordance with the Declaration of Helsinki.

### 2.5. Statistics

All statistical procedures were performed using IBM SPSS Statistics version 28. We used Kaplan–Meier non-parametric statistics to estimate the survival function from lifetime data, with the event being the patient’s death, and to plot the cumulative probability of survival at different time points. The values of median and mean survival estimates were measured to provide information about the central tendency of the survival time distribution. The value of median survival time is less sensitive to extreme values or outliers than the mean value and indicates the time by which half of the patients had died. The value of mean survival time provided an average duration for the entire study population. We also used log-rank (Mantel–Cox test) to compare the survival distributions of grouping variables: sex, age stratum (greater than or equal to/less than 65 years), and clinical subtype of ALS (spinal or bulbar). To evaluate the effect of age, sex, ALS subtype and anthropometric/biochemical nutritional markers on patient mortality at baseline and during the first three months of PEG, we fitted a Cox regression. Cox regression analysis allowed us to estimate hazard ratios, a measure of the effect of each predictor variable on mortality, considering censoring and varying follow-up times. The significance of the hazard ratios was assessed using the Wald test. Finally, linear regression analysis was used to assess whether the evolution of anthropometric/biochemical markers during the first three months of PEG was dependent on patient sex or age. All statistical test results were considered significant if the *p*-value was less than 0.05.

### 2.6. Data Availability

The datasets generated and analyzed during the current study are not publicly available due to clinical data from the hospital requiring authorization for consultation. Still, they are available from the corresponding author on reasonable request.

## 3. Results

### 3.1. Subjects

The study’s participants included 150 patients diagnosed with ALS according to the revised El Escorial or Awaji criteria who underwent PEG tube placement. The study population comprised 73 men and 77 women, with ages ranging from 32 to 88 years (mean: 66.1 [95% CI: 64–68] years; median: 67 years [IQA: 15]). Most patients (86) were elderly (65 years or older). Conversely, only 64 patients were younger adults (under 65 years). Table 2 displays the characterization of subjects’ anthropometry and laboratory serum data.

### 3.2. ALS Clinical Subtype

From the 150 patients, the clinical subtype (spinal or bulbar) was identified in 144: 88 (61%) presented a bulbar subtype, and 56 (39%) presented a spinal subtype.

### 3.3. Survival Analysis (Kaplan–Meier)

In July 2023, 4 of the 150 patients who met the inclusion criteria were lost prior to follow-up, and 27 were still alive. All surviving patients were still PEG-fed and followed up at our Artificial Nutrition Team’s Artificial Nutrition Outpatient Clinic.

**Figure 1:**
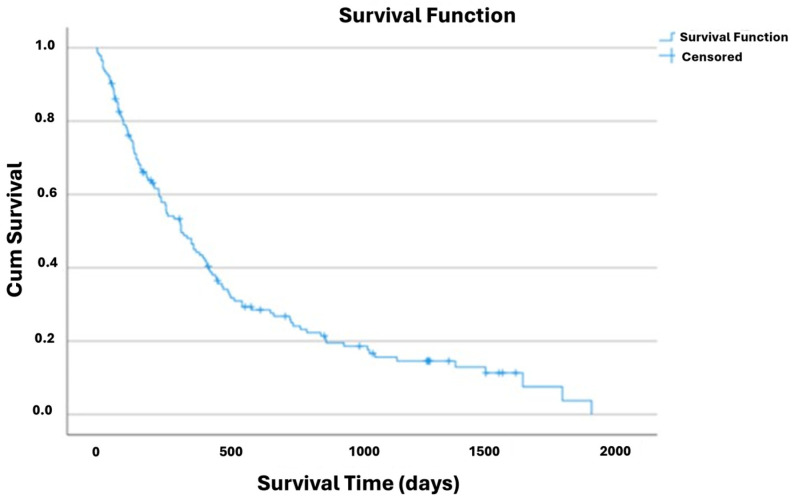
Kaplan–Meier curve of cumulative survival in patients with ALS.

After the gastrostomy endoscopic procedure, the estimated mean and median overall survival times were 527 [95% CI: 432–622] and 318 [95% CI: 236–400] days, respectively (Table A1 and Figure 1).

**Figure 2:**
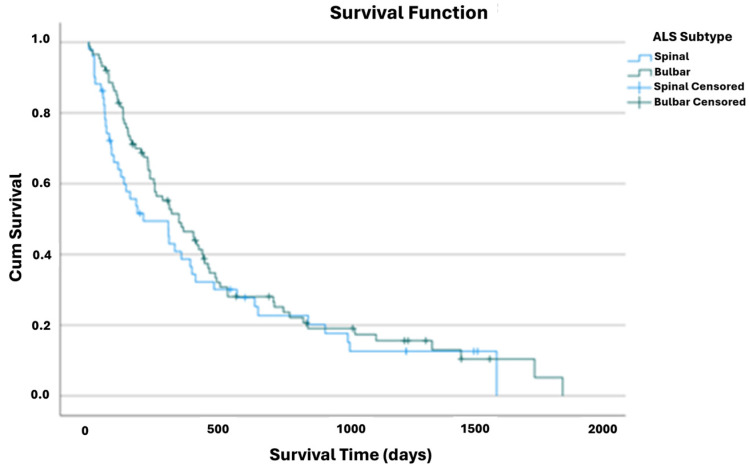
Kaplan–Meier curve of cumulative survival comparing ALS subtype group.

The bulbar ALS subtype presented a higher mean survival time 555 [95% CI: 431–679] (Table A2) after the gastrostomy. However, the difference to the spinal subtype (mean 464 [95% CI: 314–615]) was not significant (*p* = 0.277, Log Rank test), even when stratified by sex or age (Figure 2).

**Figure 3:**
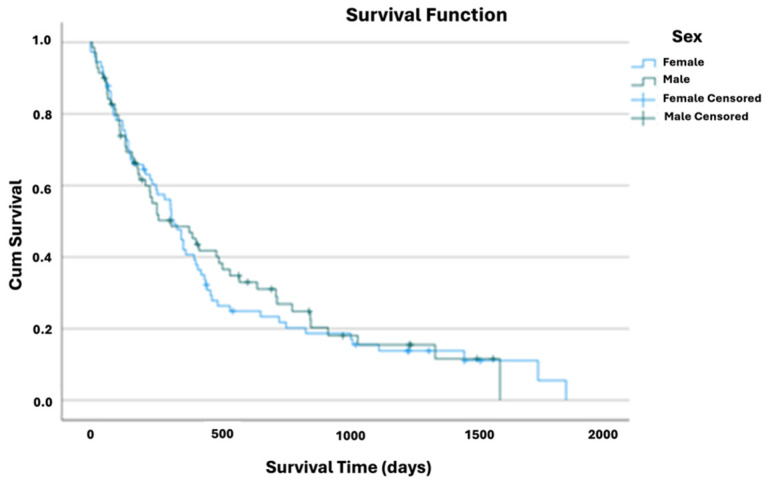
Kaplan–Meier curve of cumulative survival comparing ALS patients’ sex.

Male patients presented, on average, higher survival times (mean: 530 [95% CI: 398–662)] than females (mean: 518 [95% CI: 387–649]. Nevertheless, these survival time differences were insignificant (*p* = 0.785, Log Rank test) (Table A3 and Figure 3), even when stratifying for ALS subtype or age.

**Figure 4:**
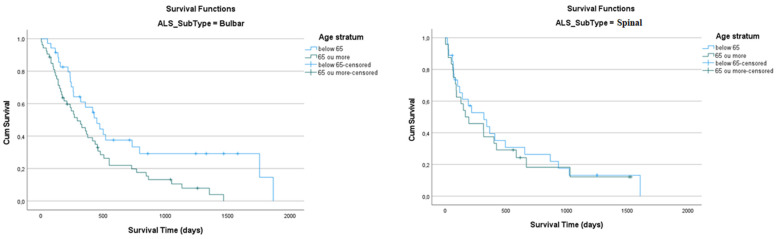
Kaplan–Meier curve of cumulative survival comparing ALS subtype at stratum ages.

Older patients, as expected, showed lower values of mean (442 [95% CI: 342–542]) and median (291 [95% CI: 205–378] survival times. However, the Log Rank test failed to validate the difference (*p* = 0.086, Log Rank test), except within the bulbar subtype (*p* = 0.013, Log Rank test) (Table A4 and Table A5 and Figure 4).

### 3.4. Cox Regression Analysis

The Cox regression evaluated three groups of predictor variables: the first included the patient’s age and sex. The second group consisted of a single variable (ALS Subtype) characterizing the ALS subtype as spinal or bulbar. The last group included baseline (at PEG tube placement) nutritional and laboratory variables (BMI, MUAC, MAMC, TSF, albumin, transferrin, cholesterol, hemoglobin) and the corresponding ones regarding the change between T0 and T3 follow-ups (MUAC_03, BMI_03, MAMC_03, TSF_03, Albumin_03, Transferrin_03, Cholesterol_03, Hemoglobin_03). Only ALS Subtype, MUAC_03 and MAMC_03 were significant (*p* < 0.05, Wald test), thus significantly influencing the death hazard.

The hazard ratio for ALS_Subtype was 0.447, indicating that, holding MUAC_03 and MAMC_03 constant, the hazard for death decreased significantly (*p* = 0.033, Wald test) as the ALS Subtype changed from spinal to bulbar. Therefore, compared to the spinal ALS subtype, individuals with bulbar disease had a 55.3% lower hazard of experiencing death and longer survival after the endoscopic procedure.

For the change in MUAC between T0 and T3, the hazard ratio was 0.900, indicating that, holding ALS subtype and MAMC_03 constant, the hazard of death decreases significantly (*p* = 0.007, Wald test) as MUAC_03 increases, resulting in a 10% lower hazard of death for each unit (cm) increase in MUAC_03.

Finally, increased MAMC_03 was significantly associated with better survival (*p* = 0.001, Wald test). The associated hazard ratio (0.892) indicates that the risk of death decreased by approximately 11% for each unit (cm) increase (Table 3).

Table 4 summarizes clinical data, including anthropometric measurements, biochemical analyses, and timelines for symptom onset, diagnosis, and PEG tube placement across ALS subtypes, as well as survival, age, and sex. Missing entries in the original dataset were managed by calculating means solely from the available data without applying imputation techniques.

The temporal dynamics between the onset of symptoms, diagnosis, and PEG tube placement in patients with ALS were examined. The analysis compared the average intervals (in days) among symptom onset, diagnosis, and PEG tube placement across various nutritional and biochemical parameters. The patients were stratified by ALS subtype (spinal or bulbar), mortality within three months or later, age groups (below 65 or equal to or above 65 years of age), and sex (female or male). A minor disagreement between the following data are due to some patients’ data missing regarding the beginning of symptoms and diagnosis, but it does not hinder a clear view of disease evolution.

The average time from symptom onset to PEG tube placement was 1419 days (*n* = 105), while the average time from diagnosis to PEG tube placement was 570 days (*n* = 149). The average time from symptom onset to diagnosis was 888 days (*n* = 104).

When stratified by ALS subtype, the average time from symptom onset to PEG tube placement was 1430 days in the spinal subtype and 1394 days in the bulbar subtype. The mean average time from diagnosis to PEG tube placement was 728 days in the spinal subtype and 467 days in the bulbar subtype. The mean average time from symptom onset to diagnosis was 689 days in the spinal subtype and 1010 days in the bulbar subtype.

When stratified by mortality within three months or more, the time from symptom onset to PEG tube placement was a mean average of 947 days in patients who died within three months and 1534 days in patients who died in a period superior to three months. The time from diagnosis to PEG tube placement was, on average, 440 days in patients who died within three months and 598 days in patients who died in a period superior over to three months. The average time from symptom onset to diagnosis was 556 days in patients who died within three months and 975 days in patients who died in a period superior to three months.

When stratified by age groups, the time from symptom onset to PEG tube placement was found to present a mean of 1165 days in patients below 65 years of age and 1624 days in patients aged 65 years or above. Similarly, the time from diagnosis to PEG tube placement was observed to be a mean of 649 days in patients below 65 years of age and 508 days in patients aged 65 years presented determined to be a mean of 556 days in patients below 65 years of age and 1151 days in patients aged 65 years or above.

When stratified by sex, the mean times from symptom onset to PEG tube placement were 1751 days for females and 1093 days for males. From diagnosis to PEG tube placement, 519 days for females and 625 days for males, and from symptom onset to diagnosis, 1233 days for females and 542 days for males.

### 3.5. Anthropometry and Laboratory Assessment at T0

According to NRS2002 [25], all patients had nutritional risk and a significant reduction in food intake. Although the GLIM (Global Leadership Initiative on Malnutrition) [26] criteria for malnutrition were not used, given that the beginning of this series is before its definition, the majority of patients presented anthropometric criteria of malnutrition.

BMI was obtained in 136 patients. For 13 patients, BMI was estimated using the Powell–Tuck and Hennessy regression equations. BMI ranged from 14.40 kg/m^2^ to 24.80 kg/m^2^ (mean: 22.92 kg/m^2^; median: 22.10 kg/m^2^). The classification was performed according to age using the Lipschitz classification [18]. Under this classification, 50 patients displayed low BMI. At ages lower than 65, 12 patients displayed a low BMI (<18.5 kg/m^2^), and with an age of 65 or more, 41 patients displayed a low BMI of <22 kg/m^2^ (Table 2). Compared with the Frisancho criteria [19], 72 of 77 patients presented a low MUAC, all presented low TSF, and 72 of 77 presented a low MAMC.

On the day of the PEG procedure, not all laboratory tests were obtained in all patients. Nonetheless, 15 out of 137 patients presented low serum Albumin, 41 out of 131 presented low serum Transferrin, 36 out of 131 presented low serum total cholesterol and 44 out of 106 presented low hemoglobin.

### 3.6. Safety

No major gastrostomy-related problems were reported or identified in any patient during follow-up.

#### Age/Sex vs. Nutritional Status Maintenance 

Linear regression results exploring MUAC_03, BMI_03, MAMC_03, TSF_03, Albumin_03, Transferrin_03, Cholesterol_03, and Hemoglobin_03 dependence on age or sex returned three meaningful regressions, all regarding age (Table A1, Table A2 and Table A3). Our results suggest that MUAC_03 (−0.137 coefficient for age, *p* = 0.035), Albumin_03 (−0.045 coefficient for age, *p* = 0.029) and Hemoglobin_03 (−0.379 coefficient for age, *p* = 0.048), values decreased significantly with increasing age, pointing out advanced age as a risk factor for an unfavorable nutritional status.

## 4. Discussion

To our knowledge, this study represents the largest effort to date focusing on PEG-fed ALS patients, including 150 individuals from a single center. Given the rarity of ALS, these findings provide significant insights into the nutritional challenges associated with the disease. Although the nature of ALS and the diversity of the patient group pose inherent challenges, our sample spans ages from 32 to 88, with a majority of patients aged 65 or older alongside a considerable number of younger adults, reflecting the disease’s epidemiology. To optimize data collection under the existing constraints, we employed the largest convenient sample available, ensuring that a sufficient number of events were observed to support a robust Kaplan–Meier survival analysis. While there is no fixed minimum sample size for such analyses, the method’s reliability depends on the number of events rather than the overall sample size, and the narrow confidence intervals of the Kaplan–Meier curves further confirm the precision of our survival estimates.

Median survival after the endoscopic procedure was slightly inferior to a single year, but mean survival was circa one and a half years, reflecting some longer survival patients. Interestingly, bulbar sub-type patients live longer after the PEG tube placement. We believe that this can occur because spinal sub-type patients without dysphagia during a long period of disease evolution tend to be referred to PEG at a more advanced moment of the disease, with worse nutritional status deterioration. These patients would likely benefit from early PEG placement and a prolonged period of mixed oral and enteral feeding, potentially mitigating the accelerated nutritional decline often observed in ALS patients. These results are consistent with sugestions from other studies, such as Borghero G. et al. (2024) [27]. Notably, less than 10% of patients died within the first three months of PEG feeding. However, the small number of early deaths is still higher than we would have wished.

As expected, anthropometric and laboratory data of our patients, observed on the day of the endoscopic procedure, reflects the compromised nutritional status that results from disease progression, oral feeding difficulties, and inflammatory status. We found a mean BMI of 22.10 Kg/m^2^, which is lower than the recommended range by ESPEN guidelines, underscoring the need for timely gastrostomy. Patients with ALS, particularly those with the bulbar subtype, may exhibit poor oral intake, leading to nutritional deterioration. Our multidisciplinary team engages with the patients’ families to discuss the various approaches, considering that all patients have an estimated survival longer than one month at admission, aligning with PEG tube placement recommendations [1].

With a mean age of 66 years and a median of 67 years, our study included predominantly older adults, highlighting the challenges of nutritional status in this age group—particularly among the older ALS patients, who exhibited worse anthropometric and laboratory results. Furthermore, the findings reported by López-Gómez et al. (2021) corroborate our results, demonstrating that older ALS patients tend to experience a more pronounced deterioration in nutritional parameters, thereby reinforcing the need for targeted intervention strategies for this group [28].

Anthropometric parameters at admission revealed poor nutrition, with over 90% of patients displaying malnutrition indicators. Arm anthropometric data (MUAC, TSF, and MAMC) indicated malnutrition, with TSF highlighting more malnourished patients than MAMC. These results suggest a preferential use of fat reserves, an adequate metabolic adaptation. Considering the anthropometric parameters presented and given that these ALS patients have an average age of 66 years, it is expected that they are at a higher risk of age-related sarcopenia [29,30].

Laboratory parameters at admission were mostly normal (in more than 60% of cases), except for hemoglobin in male patients. This parameter is often associated with poor nutritional status, but it suffers from several diverse influences and, in our study, presented no statistical significance on survival [31].

Only MUAC and MAMC significantly affected the risk of death and improvements of these parameters during the first three PEG-feeding months correlated with better survival. One unit (cm) gain in MUAC and MAMC resulted in a 10% and 11% reduction in the risk of death, respectively, highlighting the role of PEG in achieving better nutrition, increasing lean body mass and, potentially, overall survival. Although arm anthropometry has not been specifically studied in ALS [16], our experience with over 1000 neurological patients with gastrostomy correlates adequately with BMI [17].

Compared to Son et al. (2024), our study not only confirms the association between improved nutritional parameters and prolonged survival in ALS patients undergoing PEG placement but also highlights several novel aspects [11]. Unlike the earlier work, which focused primarily on overall survival trends, our research incorporates a more comprehensive set of anthropometric and biochemical markers—such as MUAC and MAMC changes over time—to better elucidate their prognostic value. Additionally, our study examines detailed time intervals from symptom onset to diagnosis and subsequent PEG placement, offering new insights into the temporal dynamics of nutritional decline in this population. These differences underscore the originality of our approach and provide a more nuanced understanding of how early nutritional intervention may influence survival outcomes, thereby contributing valuable data to the evolving clinical management of ALS.

Other anthropometric and laboratory data presented no impact on survival time after PEG, aligning with the degenerative nature of ALS, where the primary therapeutic objective is to reduce disease progression and its impact. Nutritional support should focus on preserving nutrition and, if possible, slowing the lean mass loss [32].

Notably, the present study supports the concept that PEG-fed patients whose anthropometric parameters improve may have longer survival. This finding suggests that starting an early PEG enteral feeding may extend the window for improving nutritional status, as the disease may still be in a less advanced stage.

The ESPEN guidelines recommend that discussions regarding tube feeding in patients with ALS should be initiated at the outset and conducted on a regular basis interval as the disease progresses. This proactive approach is essential for addressing evolving problems that can compromise swallowing safety and efficacy. In our experience, impairment of swallowing may progress very rapidly, and the decision to consider gastrostomy should be triggered by the earliest sign of dysphagia, prolonged meal duration, weight loss, compromised respiratory function, choking risk, and before the onset of weight loss and significant respiratory impairment [1]. A comprehensive discussion of the benefits of enteral nutrition through the natural history of ALS, is paramount for patients and their families or caregivers. In our experience, patients are frequently referred to gastrostomy at an advanced stage of ALS evolution, with substantial weight loss and a marked reduction in oral intake. This delay can be attributed to several factors, including a delayed diagnosis, inadequate follow-up by dietitians, and the patient’s extended period of decision to adopt PEG, but it should be avoided, as it can lead to irreversible nutritional deterioration.

As expected, the time intervals from the onset of symptoms to gastrostomy and from diagnosis to gastrostomy are shorter in the bulbar subtype than in the spinal subtype because the primary complications are related to oral intake and swallowing. However, the interval between the initial symptoms and diagnosis was longer in the bulbar subtype, which might be attributed to more complex diagnostic procedures or a tendency for patients to underestimate the severity of their symptoms. Furthermore, the findings reported by Wei-Ming Su et al. (2021) are in line with these observations, noting similar differences in the timing of events, though without explicitly linking the longer diagnostic interval to diagnostic challenges [33].

Concerning age and sex, the time between the initial symptoms and gastrostomy and between the initial symptoms and diagnosis is more extended in older patients and females. This may be attributed to older patients being less inclined to seek medical attention as promptly as younger patients. It is hypothesized that females may be more likely to delay seeking medical attention, prolonging the duration of their symptoms. In contrast, when we examine the interval between diagnosis and gastrostomy, younger and male patients exhibit longer times, which may be attributed to their tendency to be more reluctant to accept gastrostomy.

Considering these findings, it is imperative that gastrostomy be performed as promptly as possible to try to achieve optimal outcomes and enhance survival rates.

Unexpectedly, the bulbar ALS subtype showed a longer survival time after the gastrostomy with a 55.3% lower hazard of death after gastrostomy than the spinal subtype, contrary to general findings in the literature [34]. This anomaly may be due to the higher proportion of patients with the bulbar subtype referred early, without complications such as severe malnutrition. Although the time from symptom onset to gastrostomy is not recorded in many files, the team’s experience is consistent with an earlier gastrostomy in patients with the bulbar subtype. Conversely, patients with the spinal subtype presented with nutritional impairment at a more advanced stage of the disease. The bulbar ALS subtype may benefit from earlier enteral support, allowing nutritional status to be maintained for longer. In fact, although the longer survival after the gastrostomy procedure of bulbar subtype patients to the spinal subtype was not significant (*p* = 0.277, Log Rank test), the Cox Regression Analysis indicated that the hazard for death decreased significantly (*p* = 0.033, Wald test) for bulbar subtype patients. Patients with the spinal subtype appear to be referred to gastrostomy after a long period of progressive reduction in oral intake, in most cases when they reach severe malnutrition. In our team’s experience, patients often progressively reduce oral intake for months but consider themselves to have normal or near-normal meals. Age is also an important factor, and older patients, particularly those of the bulbar subtype, presented lower mean and median survival times.

The study monitored major PEG-related complications during follow-up, emphasizing precautionary measures for caregivers. Fortunately, no major complications were detected or reported. We believe that the reduced mobility of these patients reduces the risk of major PEG complications, and, in our team experience, this minor incidence of severe complications is similar to several other neurological disorders that progress with reduced mobility.

The present study is limited by the absence of a control group (due to ethical considerations), some incomplete data, and the difficulties encountered in data collection during the COVID-19 pandemic.

There were some inconsistencies in the calculated values regarding the time intervals between symptom onset, diagnosis, and PEG tube placement. Not all patients provided all data, and the number of patients used to calculate the time intervals between symptom onset, diagnosis, and endoscopic gastrostomy procedure varied slightly. Nevertheless, we believe these minor variations do not affect the comprehension of the global picture. Despite these limitations, the overall trends offer valuable insights into ALS patients’ clinical profiles and treatment timelines. Future studies should aim for more comprehensive data collection to strengthen the reliability of these findings.

Furthermore, it is essential to investigate the influence of PEG feeding on the quality of life of patients and caregivers to gain a comprehensive understanding of this subject. This field requires further research, mainly through more extensive, multi-center studies.

## 5. Conclusions

Endoscopic gastrostomy is a safe procedure in ALS patients with no major complications. Stratification by ALS subtype and age revealed interesting trends, with the bulbar phenotype showing longer PEG-fed survival than the spinal phenotype. Age was a key factor influencing nutrition, with older patients showing greater MUAC, albumin, and hemoglobin decreases. These findings highlight the need for close nutritional monitoring in older ALS patients.

Although our cohort demonstrated a mean survival of 17 months following PEG tube placement, many patients were already malnourished at the moment of the endoscopic gastrostomy, and some died shortly thereafter. Our findings strongly suggest that survival could be improved with earlier nutritional intervention. In many patients, PEG feeding resulted in MUAC and MAMC improvements and is associated with prolonged survival. Each unit increase in MUAC and MAMC was linked to a 10% and 11% reduction in mortality risk, respectively. Our results advocate the use of MUAC and MAMC as prognostic markers and monitoring tools in ALS care. PEG should be considered early, as a timely intervention may optimize nutritional status and improve outcomes.

## Figures and Tables

**Table 1:** Body mass index (BMI) classification according to age.

	Low	Normal	High
<65 Years	<18.5 kg/m^2^	≥18.5 and <25 kg/m^2^	≥25 kg/m^2^
≥65 Years	<22 kg/m^2^	≥22 and <27 kg/m^2^	≥27 kg/m^2^

**Table 2:** Anthropometry and biochemical characterization of subjects at T0.

Sample Anthropometric Characterization				
	Total (*n* = 136)	Male (*n* = 67)	Female (*n* = 69)	Total Mean
BMI	50 Low (36.7%)	24 Low (35.8%)	26 Low (37.7%)	22.92 kg/m^2^
	57 Normal (41.9%)	30 Normal (44.7%)	27 Normal (39.1%)	
	29 High (21.4%)	13 High (19.5%)	16 High (23.2%)	
	Total (*n* = 77)	Male (*n* = 35)	Female (*n* = 42)	
MUAC	72 Low (93.5%)	31 Low (88.6%)	41 Low (97.6%)	
	5 Normal (6.5%)	4 Normal (11.4%)	1 Normal (2.4%)	
	Total (*n* = 74)	Male (*n* = 34)	Female (*n* = 40)	
TSF	74 Low (100%)	34 Low (100%)	40 Low (100%)	
	0 Normal (0%)	0 Normal (0%)	0 Normal (0%)	
	Total (*n* = 77)	Male (*n*= 35)	Female (*n* = 42)	
MAMC	72 Low (93.5%)	31 Low (88.6%)	41 Low (97.6%)	
	5 Normal (6.5%)	4 Normal (11.4%)	1 Normal (2.4%)	
Laboratory serum data				
	Total (*n* = 137)	Male (*n* = 67)	Female (*n* = 70)	Total Mean
Albumin	15 Low (10.9%)	5 Low (7.5%)	10 Low (14.3%)	4.14 g/dL
	122 Normal (89.1%)	62 Normal (92.5%)	60 Normal (85.7%)	
	Total (*n* = 131)	Male (*n* = 65)	Female (*n* = 66)	Total Mean
Transferrin	41 Low (31.3%)	21 Low (32.3%)	20 Low (30.3%)	215.6 mg/dL
	90 Normal (68.7%)	44 Normal (67.7%)	46 Normal (69.7%)	
	Total (*n* = 131)	Male (*n* = 65)	Female (*n* = 66)	Total Mean
Total Cholesterol	36 Low (27.5%)	23 Low (35.4%)	13 Low (19.7%)	185.7 mg/dL
	95 Normal (72.5%)	42 Normal (64.6%)	53 Normal (80.3%)	
	Total (*n* = 106)	Male (*n* = 52)	Female (*n* = 54)	Total Mean
Hemoglobin	44 low (41.5%)	31 Low (62%)	13 Low (24.1%)	12.1 g/dL
	62 Normal (58.5%)	21 Normal (38%)	41 Normal (75.9%)	

BMI—body mass index; BMI classification according to age, <65 y, low BMI is <18.5 kg/m^2^, normal BMI is between 18.5 kg/m^2^ and <25 kg/m^2^, and high BMI is ≥25 kg/m^2^; ≥65 y, low BMI is <22 kg/m^2^, normal BMI is between 22 kg/m^2^ and <27 kg/m^2^, and high BMI is ≥27 kg/m^2^; (MUAC)—mid-upper arm circumference <90% low, ≥90–110% normal; (TSF)—tricipital skinfold results, <90% low, ≥90–110% normal and (MAMC)—mid-arm muscle circumference <90% low, ≥90–110% normal; albumin < 3.5 g/dL (low), transferrin < 200 mg/dL (low), total cholesterol < 160 mg/dL (low); hemoglobin—Male 14–18 g/dL (normal), Female 12–16 g/dL (normal).

**Table 3:** Factors for survival time.

	HR	95% CI Lower	Upper	Sig.
ALS Subtype	0.447	0.214	0.935	**0.033**
MUAC_03	0.900	0.833	0.972	**0.007**
MAMC_03	0.892	0.831	0.957	**0.001**

HR: hazard ratio; ALS: amyotrophic lateral sclerosis; MUAC: mid-upper arm circumference; MAMC: mid-arm muscle circumference; CI: confidence interval; Sig: *p*-value. Bold results indicate significant differences.

**Table 4:** Clinical data, onset of symptoms, diagnosis, and PEG tube placement by ALS subtype, survival, age, and sex.

	ALS Subtype				Dead at 3 Months				Age Stratum				Sex			
	Spinal		Bulbar		No		Yes		Below 65		65 or More		Female		Male	
	*n*	Mean (SD)	*n*	Mean (SD)	*n*	Mean (SD)	*n*	Mean (SD)	*n*	Mean (SD)	*n*	Mean (SD)	*n*	Mean (SD)	*n*	Mean (SD)
BMI (Kg/m^2^)	48	23.3 (5.7)	83	22.8 (5.5)	106	22.8 (4.4)	24	23.9 (9.2)	60	23.6 (5.6)	76	22.4 (5.3)	69	23.5 (6)	67	22.4 (4.9)
MAMC (cm)	55	25.2 (62.7)	90	13.2 (11.6)	**117**	**15.8 (32.65)**	**27**	**35.8 (74.4)**	67	18.5 (41)	83	19.5 (45.1)	77	15.3 (31.1)	73	22.9 (52.9)
MUAC (cm)	22	66.1 (86.8)	52	26.9 (3.6)	61	34.1 (39.8)	14	72.3 (92.5)	35	38.9 (51)	42	42.3 (57.3)	42	32.3 (37.8)	35	84.5 (12)
TSF (mm)	20	10.9 (4.95)	52	12.9 (6.03)	58	12.8 (5.73)	14	10.1 (5.6)	33	12.4 (6.9)	41	12.4 (4.6)	40	14.4 (5.8)	**34**	**80.1 (38.5)**
Albumin (g/dL)	49	4.1 (0.54)	83	4.2 (0.5)	**108**	**4.2 (0.5)**	**24**	**3.8 (0.6)**	**63**	**4.3 (0.5)**	**74**	**4.0 (0.5)**	70	4.1 (0.5)	67	4.21 (0.47)
Transferrin (mg/dL)	47	212 (38)	79	216 (41)	103	217 (41)	23	204 (45)	59	216 (35)	72	215 (46)	66	215 (42)	65	216 (42)
Total Cholesterol (mg/dL)	46	188 (44)	80	185 (39)	102	187 (39)	24	177 (47)	61	193 (40)	70	179 (39)	66	190 (41)	65	181 (40)
Hemoglobin (g/dL)	36	125.1 (33.5)	66	124.5 (31.7)	84	126.1 (32.5)	18	116.8 (30.2)	51	126.6 (33.5)	55	123.1 (30)	54	121.3 (24.6)	52	128.4 (37.4)
Time Syn_PEG (days)	41	1430 (1152)	63	1394 (5233)	79	1534 (4685)	20	947 (934)	47	1165 (949)	58	1624 (5470)	52	1751 (5740)	53	1093 (1079)
Time Diag_PEG (days)	**55**	**728 (655)**	**89**	**467 (449)**	116	598 (565)	27	440 (479)	66	649 (610)	83	508 (478)	77	519 (453)	72	625 (624)
Time Syn_Diag (days)	41	689 (825)	62	1010 (5179)	78	975 (4619)	20	556 (668)	46	556 (594)	58	1151 (5366)	52	1233 (5647)	52	542 (732)

BMI—body mass index; *n*—sample size; (MUAC)—mid-upper arm circumference; (TSF)—tricipital skinfold; (MAMC)—mid-arm muscle circumference; Time sin_PEG—time between first symptoms to PEG tube placement; Time Diag_PEG—time between diagnostic to PEG tube placement; Time Sin_Diag—time between first symptoms to diagnostic. Bold results indicate significant differences (*p*-value < 0.05; Student *t* test) between pairs of subcategories.

## Data Availability

The data presented in this study are available upon request from the first author.

## Appendix A

**Table A1:** Means and medians for global sample survival time (days).

Mean Survival ^a^	Std. Error	95% CI Lower Bound	Upper Bound	Median Survival	Median Survival	95% CI Lower Bound	Upper Bound
527	49	432	622	318	42	236	400

^a.^ Estimation is affected by extended survival times and censored cases; Std. Error: Standard Error; CI: Confidence Interval.

**Table A2:** Means and medians for Survival Time (days) comparing ALS subtype (spinal versus bulbar).

ALS Subtype	Mean Survival ^a^	Std. Error	95% CI		Median Survival	Std. Error	95% CI		Log Rank (Mantel–Cox)	
			Lower Bound	Upper Bound			Lower Bound	Upper Bound	Chi-Square	Sig.
Spinal	464	77	314	615	216	86	48	384	1.180	0.277
Bulbar	555	63	431	679	356	60	240	473		

^a^ Estimation is affected by extended survival times and censored cases; Std. Error: Standard Error; CI: Confidence Interval; Sig.: *p*-value.

**Table A3:** Means and medians for Survival Time comparing ALS patients’ sex.

Sex	Mean Survival ^a^	Std. Error	95% CI		Median Survival	Std. Error	95% CI		Log Rank (Mantel–Cox)	
			Lower Bound	Upper Bound			Lower Bound	Upper Bound	Chi-Square	Sig.
Female	518	67	387	649	327	34	260	394	0.074	0.785
Male	530	67	398	662	317	93	136	498		

^a^ Estimation is affected by extended survival times and censored cases; Std. Error: Standard Error: CI: Confidence Interval; Sig.: *p*-value.

**Table A4:** Means and medians for Survival Time comparing ALS patients’ age stratum.

Age Stratum	Mean Survival ^a^	Std. Error	95% CI		Median Survival	Std. Error	95% CI		Log Rank (Mantel–Cox)	
			Lower Bound	Upper Bound			Lower Bound	Upper Bound	Chi-Square	Sig.
below 65	625	84	460	791	387	59	272	502	2.942	0.086
65 or more	442	51	342	542	291	44	205	378		

^a^ Estimation is affected by extended survival times and censored cases; Std. Error: Standard Error; CI: Confidence Interval; Sig.: *p*-value.

**Table A5:** Comparison of ALS patients’ survival time between age stratum for each ALS subtype.

		Log Rank (Mantel–Cox)	Sig.
Spinal	below 65	0.145	0.703
	65 ou more		
Bulbar	below 65	6.145	**0.013**
	65 ou more		

Sig.: *p*-value. Bold results indicate significant differences.

**Table A6:** Age or sex over-dependent variable MUAC.

Model		Unstandardized Coefficients		Standardized Coefficients	T	Sig.	95.0% Confidence Interval for B		Collinearity Statistics	
		B	Std. Error	Beta			Lower Bound	Upper Bound	Tolerance	VIF
1	(Constant)	7.142	4.354		1.640	0.109	−1.664	15.949		
	Age	−0.135	0.063	−0.322	−2.132	0.039	−0.263	−0.007	0.998	1.002
	Sex	−0.857	1.470	−0.088	−0.583	0.563	−3.831	2.117	0.998	1.002
(Constant)		6.901	4.298		1.606	0.116	−1.786	15.587		
Age		−0.137	0.063	−0.325	−2.176	0.035	−0.264	−0.010	1.000	1.000

**Table A7:** Age or sex over-dependent variable albumin.

Model		Unstandardized Coefficients		Standardized Coefficients	t	Sig.	95.0% Confidence Interval for B		Collinearity Statistics	
		B	Std. Error	Beta			Lower Bound	Upper Bound	Tolerance	VIF
1	(Constant)	2.891	1.476		1.959	0.054	−0.050	5.832		
	Age	−0.043	0.021	−0.239	−2.031	0.046	−0.085	−0.001	0.927	1.079
	Sex	0.195	0.506	0.045	0.385	0.702	−0.814	1.203	0.927	1.079
1	(Constant)	3.133	1.328		2.359	0.021	0.487	5.778		
	Age	−0.045	0.020	−0.251	−2.231	0.029	−0.085	−0.005	1.000	1.000

**Table A8:** Age or sex over-dependent variable hemoglobin.

Model		Unstandardized Coefficients		Standardized Coefficients	t	Sig.	95.0% Confidence Interval for B		Collinearity Statistics	
		B	Std. Error	Beta			Lower Bound	Upper Bound	Tolerance	VIF
1	(Constant)	23.987	12.427		1.930	0.060	−1.058	49.033		
	Age	−0.379	0.187	−0.293	−2.029	0.048	−0.755	−0.003	1.000	1.000
